# Association between serum albumin levels and height loss in Japanese workers: a retrospective study

**DOI:** 10.1186/s40101-023-00338-z

**Published:** 2023-09-12

**Authors:** Yuji Shimizu, Hidenobu Hayakawa, Eiko Honda, Nagisa Sasaki, Midori Takada, Takeo Okada, Tetsuya Ohira, Masahiko Kiyama

**Affiliations:** 1 Epidemiology Section, Division of Public Health, Osaka Institute of Public Health, Osaka, Japan; 2 Department of Cardiovascular Disease Prevention, Osaka Center for Cancer and Cardiovascular Diseases Prevention, Osaka, Japan; 3 Department of Epidemiology, Fukushima Medical University School of Medicine, Fukushima, Japan

**Keywords:** Albumin, Height loss, Workers, Retrospective

## Abstract

**Background:**

Height loss starting in middle age was previously shown to be associated with high cardiovascular mortality in later life. However, the factors associated with height loss remain unknown. Since low serum albumin levels are reported to be associated with high mortality caused by cardiovascular disease, they may also contribute to height loss.

**Methods:**

To clarify the association between serum albumin and height loss, we conducted a retrospective study of 7637 Japanese workers who participated in general health check-ups from 2008 to 2019. Height loss was defined as the highest quartile of height loss per year.

**Results:**

Individual with high serum concentration of albumin possess beneficial influence on preventing incidence of height loss. In both men and women, serum albumin level was significantly inversely associated with height loss. After adjustment for known cardiovascular risk factors, the adjusted odd ratio (OR) and 95% confidence interval (CI) for height loss per 1 standard deviation of albumin (0.2 g/dL for both men and women) were 0.92 (0.86, 0.98) in men and 0.86 (0.79, 0.95) in women. Even when the analysis was limited to participants without hypoalbuminemia, essentially same association was observed, with fully adjusted corresponding ORs (95%CI) of 0.92 (0.86, 0.98) in men and 0.86 (0.78, 0.94) in women.

**Conclusion:**

Independent of known cardiovascular risk factors, higher serum albumin levels may prevent height loss among Japanese workers. While several different diseases cause hypoalbuminemia, they may not be the main reasons for the association between serum albumin and height loss. Though further research is necessary, this finding may help clarify the mechanisms underlying the association between height loss and higher mortality in later life.

## Introduction

Height loss starting in middle age was previously shown to be associated with high cardiovascular mortality in later life [1]. Intervertebral disc degeneration, which is associated with narrowing of the intervertebral spaces, and vertebral fracture are both well-known causes of height loss in adults. Since low serum albumin levels were shown to be associated with high mortality [2], including that due to cardiovascular diseases [3], they may also correlate with height loss, although no studies thus far have reported this connection.

Serum albumin is synthesized in the liver and it accounts for the majority of total serum proteins. Serum albumin helps maintain colloid osmotic pressure and facilitates the transportation of many substances such as bilirubin, uric acids, free fatty acids, electrolytes, thyroxine, and numerous drugs. A variety of health conditions influence serum albumin levels, and therefore among workers who are relatively healthy, these levels may act as a marker of general health even though they cannot indicate the presence of specific diseases.

Short stature in adulthood was reported to be positively associated with cardiovascular disease [4, 5], and the incidence of cardiovascular disease was positively correlated with low serum albumin levels [6]. Known cardiovascular risk factors could act as confounding factors in the association between serum albumin levels and adult height loss in the general population.

To clarify the association between serum albumin levels and adult height loss, we conducted a retrospective study with a median follow-up duration of 3.1 years in 7637 Japanese workers who participated in general health check-ups at least twice between 2008 and 2019.

## Methods

### Study population

In 2008, the Ministry of Health, Labor, and Welfare of Japan started a program involving medical examinations specifically aimed at cardiovascular disease prevention. Height was measured at least twice during follow-up (2008–2019) to calculate height loss. The initial population in this study comprised 15,435 workers aged 40–74 years who participated in these examinations between 2008 and 2018 (baseline) at the Osaka Center for Cancer and Cardiovascular Diseases Prevention, whose Ethics Committee approved this study (Project registration code: R4-Rinri-4).

Subjects without data on drinking status (*n* = 57), total cholesterol (TC) (*n* = 1179), hemoglobin A1c (HbA1c) (*n* = 20), or albumin (*n* = 3789) at baseline were excluded from the analysis. Subjects without a height measurement from 2009 to 2019 (endpoint) were also excluded from the analysis (*n* = 2753). The remaining 7637 subjects, with a mean age of 50.5 years (standard deviation [SD], 8.1 years; range, 40–74 years), were included in the study.

### Data collection and laboratory measurements

#### Baseline data

The baseline period of the present study was 2008–2018. Trained interviewers acquired medication histories and data on smoking and drinking habits. Briefly, subjects wore stockings and light clothing during measurements of height and weight, respectively. Body mass index (BMI) was calculated as weight divided by height (kg/m^2^). Resting blood pressure was measured twice. Mean blood pressure data were used in the analysis.

Fasting blood samples were collected. Hemoglobin (Hb), TC, triglycerides (TG), high-density lipoprotein cholesterol (HDLc), HbA1c, and serum creatinine were measured using the standard procedures at the Osaka Center for Cancer and Cardiovascular Diseases Prevention. Low-density lipoprotein cholesterol (LDLc) was calculated using the Friedewald formula: LDLc = TC-(HDLc/5) mg/dL.

Between 2008 and 2012, HbA1c values were measured using the Japanese Diabetes Society (JDS) definition. Starting in 2013, HbA1c values were measured using the National Glycohemoglobin Standardization Program (NGSP) definition. The following equation, which was recently proposed by a JDS working group, was used to convert these values: HbA1c(NGSP) = HbA1c(JDS) + 0.4% [7].

The World Health Organization (WHO) guidelines state that in Asians, a high BMI is defined as ≥ 25 kg/m^2^ [8], and we adopted this definition.

The estimated glomerular filtration rate (eGFR; mL/min/1.73 m^2^) was calculated using an established equation modified recently by a working group of the Japanese Chronic Kidney Disease Initiative [9], as follows: 194 × (serum creatinine (enzyme method))^−1.094^ × (age) ^−0.287^ (× 0.739 for women). Chronic kidney disease was defined as eGFR < 60 mL/min/1.73 m^2^.

Hypertension was defined as systolic blood pressure ≥ 140 mmHg, diastolic blood pressure ≥ 90 mmHg, or use of anti-hypertensive medication. Dyslipidemia was defined as TG ≥ 150 mg/dL, LDLc ≥ 140 mg/dL, HDLc < 40 mg/dL, or use of lipid-lowering medication. Diabetes was defined as HbA1c (NGSP) ≥ 6.5% or use of glucose-lowering medication.

### Endpoint data

Height was measured during the endpoint period (2009–2019). A participant was considered to have height loss if he or she was in the highest quartile of height loss per year, as in a previous study [10].

### Statistical analysis

Sex-specific characteristics of the study participants were analyzed according to tertiles of serum albumin levels. Age is expressed as mean ± SD. Daily drinking, current smoker status, hypertension, high BMI, dyslipidemia, diabetes, and chronic kidney disease are presented as percentages.

Logistic regression was used to calculate odd ratios (ORs) and 95% confidence intervals (CIs) to determine associations between serum albumin and other variables. Two adjustment models were used. The first adjusted only for age (age-adjusted model). The second (multivariable model) also included established parameters of cardiovascular risk that are known to be confounding factors, specifically drinking status (none, often, daily), smoking status (never, former, current), hypertension (no versus yes), high BMI (no versus yes), diabetes (no versus yes), dyslipidemia (no versus yes), and chronic kidney disease (no versus yes).

All statistical analyses were performed with SAS for Windows (version 9.4; SAS Inc., Cary, NC, USA); *p* values of < 0.05 were regarded as statistically significant.

## Results

### Subject characteristics by albumin level

Table 1 shows the characteristics of the study population stratified by tertiles of albumin levels. In men, the albumin level was significantly positively associated with high BMI and dyslipidemia, and inversely associated with age, current smoking status, and hypertension. In women, the albumin level was positively associated with dyslipidemia and inversely associated with high BMI.

**Table 1:** Subject characteristics

	Albumin tertile levels			*p*
	T1 (Low)	T2 (Middle)	T3 (High)	
Men				
No. at risk	1869	1712	1423	
Age, years	53.5 ± 7.9	50.5 ± 8.1	48.5 ± 8.0	< 0.001
Daily drinker, %	25.3	23.6	22.6	0.185
Current smoker, %	36.8	32.7	28.7	0.015
Hypertension, %	44.6	33.4	31.4	< 0.001
High BMI, %	31.6	34.2	36.4	0.015
Dyslipidemia, %	47.9	52.9	59.0	< 0.001
Diabetes, %	8.5	6.8	9.1	0.049
CKD, %	11.8	10.2	10.2	0.191
Women				
No. at risk	716	957	960	
Age, years	49.8 ± 8.0	49.2 ± 7.4	49.5 ± 7.9	0.301
Daily drinker, %	10.2	9.2	11.6	0.233
Current smoker, %	11.7	10.4	10.3	0.606
Hypertension, %	19.8	17.1	16.7	0.208
High BMI, %	18.4	14.2	13.2	0.009
Dyslipidemia, %	32.8	32.7	38.5	0.011
Diabetes, %	2.0	2.0	1.9	0.984
CKD, %	11.9	12.7	14.3	0.331

*BMI* body mass index, *CKD* chronic kidney disease. Age: mean ± standard deviation. Albumin tertile levels for men were < 4.5 g/dL for T1 (low), 4.5–4.6 g/dL or T2 (middle), and ≥ 4.7 g/dL for T3 (high), and for women the corresponding values were < 4.4 g/dL, 4.4–4.5 g/dL, and ≥ 4.6 g/dL

### Association between height loss and serum albumin level

In both men and women, the serum albumin level was significantly inversely associated with height loss (Table 2). These associations were unchanged even after further adjustment for known cardiovascular risk factors. The fully adjusted ORs and 95% CIs for height loss per SD increment of serum albumin (0.2 g/dL for both men and women) were 0.92 (0.86, 0.98) in men and 0.86 (0.79, 0.95) in women. To avoid the influence of hypoalbuminemia (< 3.8 g/dL) [11], the association between height loss and serum albumin among participants without hypoalbuminemia was also evaluated and was found to be essentially the same, with fully adjusted corresponding ORs (95%CIs) of 0.92 (0.86, 0.98) in men (*n* = 4993) and 0.86 (0.78, 0.94) in women (*n* = 2631).

**Table 2:** Odds ratios (OR) and 95% confidence intervals (CI) for height loss in relation to albumin level

	Albumin tertile levels			*p*	1-SD increment of albumin
	T1 (Low)	T2 (Middle)	T3 (High)		
Men					
No. of participants	1869	1712	1423		
No. of participants with height loss (percentage)	525 (28.1)	415 (24.2)	310 (21.8)		
Age-adjusted ORs	Reference	0.91 (0.78, 1.06)	0.84 (0.72, 0.998)	0.043	0.92 (0.86, 0.98)
Multivariable ORs	Reference	0.91 (0.78, 1.06)	0.85 (0.72, 1.01)	0.052	0.92 (0.86, 0.98)
Women					
No. of participants	716	957	960		
No. of participants with height loss (percentage)	209 (29.2)	233 (24.3)	216 (22.5)		
Age-adjusted ORs	Reference	0.80 (0.64, 1.00)	0.71 (0.57, 0.89)	0.003	0.86 (0.79, 0.94)
Multivariable ORs	Reference	0.81 (0.65, 1.02)	0.71 (0.57, 0.89)	0.003	0.86 (0.79, 0.95)

*Multivariable ORs*: further adjusted for age, drinking status, smoking status, high BMI, hypertension, diabetes, dyslipidemia, and chronic kidney disease. *Height loss*: the highest quartile of height loss per year. Albumin tertile levels for men were < 4.5 g/dL for T1 (low), 4.5–4.6 g/dL or T2 (middle), and ≥ 4.7 g/dL for T3 (High), and for women the corresponding values were < 4.4 g/dL, 4.4–4.5 g/dL, and ≥ 4.6 g/dL. The 1-standard deviation increment of albumin was 0.2 g/dL in both men and women

### Sensitivity analysis

To assess sensitivity, we again analyzed the association between serum albumin and height loss, this time with height loss defined as the highest tertile of height loss per year rather than the highest quartile. We obtained essentially the same results. In the multivariable model, the ORs for height loss were 0.93 (0.87, 0.99) in men and 0.89 (0.82, 0.97) in women.

## Discussion

The major finding of the present study is that in both male and female Japanese workers in the general population, serum albumin was significantly inversely associated with height loss.

A previous study in European men reported a modest impact of several factors, including serum albumin, on the association between marked height loss and increased risk of all-cause mortality [12].

This is unsurprising, since height loss beginning in middle age was previously found to be associated with high subsequent mortality due to cardiovascular disease [1], and low serum albumin levels were reported to be associated with high mortality due to cardiovascular disease [3]. However, the mechanism underlying the association between albumin level and height loss has not yet been clarified.

When we limited our analysis to participants without hypoalbuminemia, we observed essentially the same association between serum albumin and height loss as that seen in the full sample. Therefore, although several different diseases cause hypoalbuminemia, such as cirrhosis, malnutrition, nephrotic syndrome, and sepsis [13], these conditions may not have been the main explanation for the association between serum albumin and height loss observed in this study. If these diseases do strongly influence this association, their impact was eliminated when the analysis was performed among those without hypoalbuminemia.

In the present study, current smoking was inversely associated with serum albumin in men but not in women. Further, the prevalence of high BMI was positively associated with serum albumin in men but inversely associated with serum albumin in women. Since a significant inverse association between serum albumin and height loss was observed in both men and women, current smoking and high BMI are also not the primary explanation for this association.

Disc degeneration and osteoporosis may be responsible since the major causes of height loss in adults are disc degeneration (which causes intervertebral narrowing) and vertebral fracture (which is strongly associated with osteoporosis).

Inflammation is a known contributor to both intervertebral disc degeneration [14] and osteoporosis [15]. Since chronic inflammation can cause hypoalbuminemia [16], it might also underlie the inverse association between serum albumin and height loss. Smoking is a known cause of low-grade chronic inflammation. In the present study, current smoking was significantly inversely associated with serum albumin in men but not in women. The inverse association in men may be due to chronic inflammation leading to reduced serum albumin levels.

**Fig. 1:**
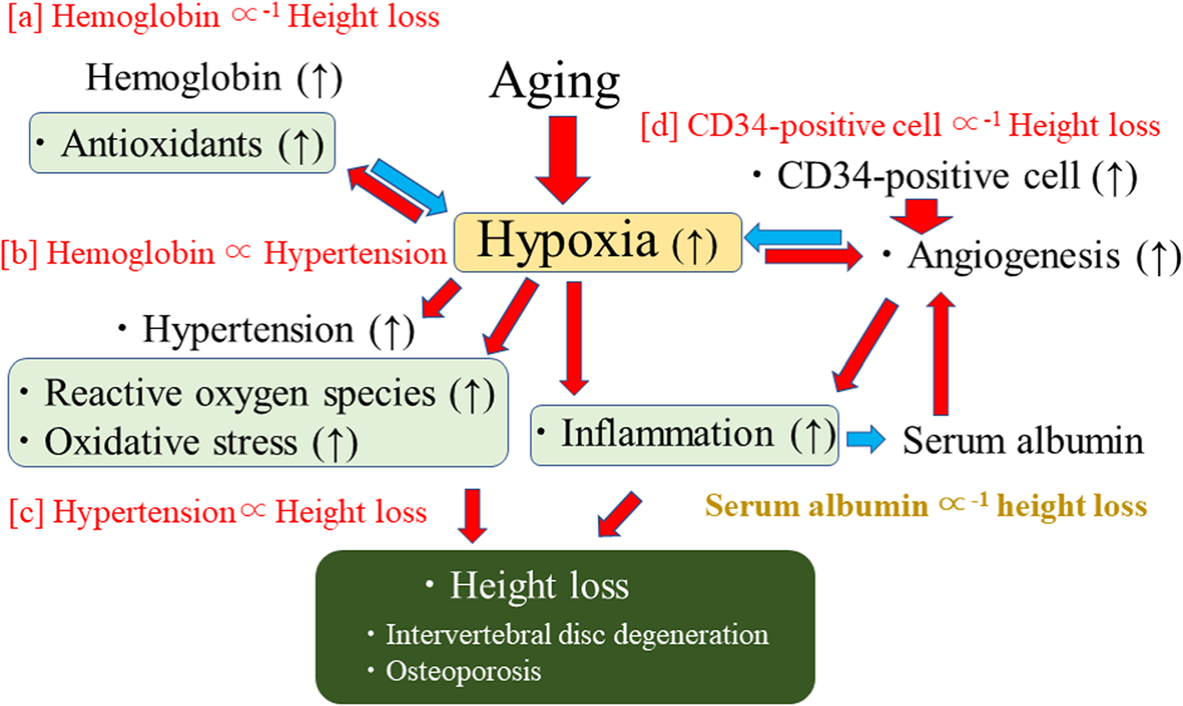
Potential mechanism underlying the association between serum albumin and height loss. Associations shown in red [a-d] were observed in the previous studies. Red arrow indicates "stimulate". Blue arrow indicates "inhibit"

There are strong connections between hypoxia, oxidative stress, and inflammation [17]. Hypoxic conditions favor the increase of reactive oxygen species and oxidative stress [17]. Hypoxia plays a key role in the regulation of immunity and inflammation [18]. Specific diseases that relate to hypoalbuminemia may not have the main explanation for the association between serum albumin and height loss. Therefore, physical condition which relates to normal process of aging that is shown in Fig. 1 might took important role on present results. Aging is the process that often accompanied by hypoxia, oxidative stress, and chronic inflammation [19]. While angiogenesis contributes to inflammation [20], it also strongly contributes to reducing hypoxia and oxidative stress [21, 22]. Angiogenesis may have played an important role in the present results. It is reportedly positively associated with intervertebral disc degeneration [23, 24] and osteoporosis [25, 26]. However, angiogenesis per se might not be a risk factor for height loss, but oxidative stress and hypoxia that causes progression of angiogenesis might act as an independent risk factor both for intervertebral disk degeneration and osteoporosis. The development of angiogenesis should have a beneficial influence on preventing height loss by reducing oxidative stress and hypoxia. Serum albumin strongly influences stem cell migration [27], which is known to contribute to angiogenesis [28]. Therefore, lower albumin levels could be associated with lower angiogenesis activity due to inhibition of stem cell migration. In fact, angiogenesis inhibitor therapies might be associated with an increased incidence of cardiovascular disease [29], which was previously shown to be the case with low serum albumin levels [6]. In addition, independent of known cardiovascular risk factors, a previous study of 363 men aged 60 to 69 years revealed a significant inverse association between the number of circulating CD34-positive cells and height loss, defined as the highest quartile of height loss per year [10] (Fig. 1d). Since CD34-positive cells play a primary role in the development of angiogenesis [30], a shortage of circulating these cells could result in reduced angiogenesis capacity and thereby increase the risk of height loss.

In addition, hypertension is induced by angiogenesis inhibitors [31], and its risk is increased by insufficient numbers of circulating CD34-positive cells [21, 22]. These facts are consistent with a previous study showing that the risk of height loss was increased by hypertension [32] (Fig. 1c) and by lower angiogenesis activity. The present study demonstrated a significant inverse association between hypertension and serum albumin in men, and even though the statistical difference did not reach significance, the inverse relationship between these two was also observed in women. Therefore, serum albumin may reflect angiogenesis activity which in turn may prevent hypertension [33, 34].

Furthermore, chronic inflammation is also positively associated with both hypertension [35] and hypoalbuminemia [16]. Chronic inflammation stimulates the progression of angiogenesis [20]. However, to reduce high levels of oxidative stress, increased angiogenesis is required. This may explain the presence of angiogenesis in vertebral disc degeneration [23, 24] and osteoporosis [25, 26]. Then, development of angiogenesis in these diseases might indicate the presence of insufficient angiogenesis. Further, the degree of angiogenesis may be insufficient to reduce oxidative stress occurring at very high levels.

Given that albumin acts as an antioxidant [36], it is possible that antioxidants play an important role in the association between serum albumin and height loss. Hypoxia is known to cause increased oxidative stress [17]. Increased hemoglobin levels help reduce oxidative stress by increasing oxygen supply. Therefore, serum albumin and hemoglobin could act together as antioxidants and as markers of general vascular health. This is partly supported by a previous study in which low levels of albumin and hemoglobin were potentially useful risk markers of physical functional decline in older adults [37]. Our previous study in Japanese workers showed that hemoglobin exerted a preventive effect on height loss [38] (Fig. 1a). However, hemoglobin was also reported to be positively associated with hypertension [39] (Fig. 1b), which is a known risk factor for height loss [32]. These studies indicate that high oxidative stress might be a common characteristic of physical conditions in which hematopoiesis is induced, since hematopoiesis might help reduce oxidative stress [40]. To clarify the mechanisms involved, further studies should investigate the combined roles of inflammation, oxidative stress, angiogenesis activity, and antioxidant activity.

This is the first study to report an independent and significant inverse association between serum albumin levels and height loss in Japanese workers. Since height loss starting in middle age may be a marker of high mortality in later life [1], the present findings may serve as a novel and effective means of estimating mortality risk.

Potential limitations of this study warrant consideration. First, in adults, height loss can be caused by vertebral fractures associated with osteoporosis and by intervertebral disc degeneration. We did not have data on these conditions so further research is necessary. Second, an accurate cutoff point to define height loss has not been established. In the present study, we used the highest quartile of height loss per year. However, our sensitivity analysis that used tertiles of height loss per year yielded essentially the same results. Chronic inflammation, angiogenesis activity, and antioxidant activity may have contributed to the present results, but we had no data on any of these. Analyses of these topics in addition to other endocrine [41, 42] and genetic factors [43, 44] are necessary to clarify the mechanisms underlying the association between serum albumin and height loss.

## Conclusion

In conclusion, serum albumin was significantly inversely associated with height loss in both male and female Japanese workers. Since height loss has been reported to be associated with high mortality, these findings may help estimate mortality risk.

## Data Availability

The datasets generated and/or analyzed during the current study are not publicly available due to ethical considerations. Qualified researchers may apply for access a minimal dataset by contacting Dr. Masahiko Kiyama, General Coordinator, at kiyama@osaka-ganjun.jp or data management staff at kenkyu_gyomu@osaka-ganjun.jp. Information regarding data requests is also available at http://www.osaka-ganjun.jp (accessed on 20 July 2022).

## Abbreviations

TC: Total cholesterol
HbA1c: Hemoglobin A1c
SD: Standard deviation
BMI: Body mass index
Hb: Hemoglobin
TG: Triglycerides
HDLc: High-density lipoprotein cholesterol
LDLc: Low-density lipoprotein cholesterol
JDS: Japanese Diabetes Society
NGSP: National Glycohemoglobin Standardization Program
WHO: World Health Organization
ORs: Odd ratios
CIs: Confidence intervals
